# Comparison of the efficacy of a commercial inactivated influenza A/H1N1/pdm09 virus (pH1N1) vaccine and two experimental M2e-based vaccines against pH1N1 challenge in the growing pig model

**DOI:** 10.1371/journal.pone.0191739

**Published:** 2018-01-30

**Authors:** Tanja Opriessnig, Phillip C. Gauger, Priscilla F. Gerber, Alessandra M. M. G. Castro, Huigang Shen, Lita Murphy, Paul Digard, Patrick G. Halbur, Ming Xia, Xi Jiang, Ming Tan

**Affiliations:** 1 The Roslin Institute and The Royal (Dick) School of Veterinary Studies, University of Edinburgh, Midlothian, United Kingdom; 2 Department of Veterinary Diagnostic and Production Animal Medicine, College of Veterinary Medicine, Iowa State University, Ames, Iowa, United States of America; 3 Division of Infectious Diseases, Cincinnati Children’s Hospital Medical Center, Cincinnati, Ohio, United States of America; 4 Department of Pediatrics, University of Cincinnati, College of Medicine, Cincinnati, Ohio, United States of America; University of South Dakota, UNITED STATES

## Abstract

Swine influenza A viruses (IAV-S) found in North American pigs are diverse and the lack of cross-protection among heterologous strains is a concern. The objective of this study was to compare a commercial inactivated A/H1N1/pdm09 (pH1N1) vaccine and two novel subunit vaccines, using IAV M2 ectodomain (M2e) epitopes as antigens, in a growing pig model. Thirty-nine 2-week-old IAV negative pigs were randomly assigned to five groups and rooms. At 3 weeks of age and again at 5 weeks of age, pigs were vaccinated intranasally with an experimental subunit particle vaccine (NvParticle/M2e) or a subunit complex-based vaccine (NvComplex/M2e) or intramuscularly with a commercial inactivated vaccine (Inact/pH1N1). At 7 weeks of age, the pigs were challenged with pH1N1 virus or sham-inoculated. Necropsy was conducted 5 days post pH1N1 challenge (dpc). At the time of challenge one of the Inact/pH1N1 pigs had seroconverted based on IAV nucleoprotein-based ELISA, Inact/pH1N1 pigs had significantly higher pdm09H1N1 hemagglutination inhibition (HI) titers compared to all other groups, and M2e-specific IgG responses were detected in the NvParticle/M2e and the NvComplex/M2e pigs with significantly higher group means in the NvComplex/M2e group compared to SHAMVAC-NEG pigs. After challenge, nasal IAV RNA shedding was significantly reduced in Inact/pH1N1 pigs compared to all other pH1N1 infected groups and this group also had reduced IAV RNA in oral fluids. The macroscopic lung lesions were characterized by mild-to-severe, multifocal-to-diffuse, cranioventral dark purple consolidated areas typical of IAV infection and were similar for NvParticle/M2e, NvComplex/M2e and SHAMVAC-IAV pigs. Lesions were significantly less severe in the SHAMVAC-NEG and the Inact/pH1N1pigs. Under the conditions of this study, a commercial Inact/pH1N1 specific vaccine effectively protected pigs against homologous challenge as evidenced by reduced clinical signs, virus shedding in nasal secretions and oral fluids and reduced macroscopic and microscopic lesions whereas intranasal vaccination with experimental M2e epitope-based subunit vaccines did not. The results further highlight the importance using IAV-S type specific vaccines in pigs.

## Introduction

Influenza A virus (IAV), a genus within the family *Orthomyxoviridae*, is a negative sense, single-stranded RNA virus. The IAV genome consists of eight segments that encode a minimum of 10 viral proteins. The glycoproteins hemagglutinin and neuraminidase are located on the surface of the virus and often used for characterization of IAV strains [1]. These glycoproteins are responsible for viral entry and subsequent viral release from the infected cells, are highly variable and are the major targets of the host humoral immune response [1].

Swine IAV (IAV-S) is considered one of the most important causes of acute respiratory diseases in U.S. swine herds. Infection of pigs with IAV-S typically manifests as an acute, highly contagious disease that often affects an entire herd in a short period of time [2]. The main clinical signs of IAV-S infection are depression, fever, anorexia, coughing and dyspnea [2]. Mortality can vary from 1–4%. In addition, IAV-S is considered a zoonotic pathogen and transmission between pigs and humans occurs on a regular basis [3,4] making IAV-S an important public health consideration. The populations of IAV found in North American pigs are very diverse and include H3N2, H1N1 and H1N2 viruses with a more limited distribution of other subtypes. During 2009, a worldwide influenza pandemic with a novel swine-derived A/H1N1/pdm09 (pH1N1) occurred in humans [5] and the virus also re-entered the pig population where it continues to spread. Commercial inactivated vaccines against pH1N1 are available to date for pigs and it has also been shown that vaccination of swine workers had a beneficial effect on minimizing IAV-S transmission between farms [6].

Vaccination, while often used in U.S. pigs, is not always effective due to the heterogeneity of circulating IAV-S strains. All of the licensed, commercial IAV-S vaccines currently available in the U.S. contain inactivated virus or subunit virus particles. Influenza virus is cultivated in eggs or cell culture and inactivated with chemical agents, such as formaldehyde or binary ethylenimine for use in vaccines [7]. There is a need for novel and universal vaccine strategies capable of cross-protection against genetically different IAV-S strains.

Recently, two polyvalent vaccine platforms based on the norovirus (Nv) capsid protein have been developed [8,9] and utilized for the development of a universal IAV-S vaccine [10,11]. The M2 protein is integrated into the viral envelope of IAV and its ion channel activity is required for efficient viral uncoating during virus invasion of cells. The M2e epitope, which is 23 amino acid residues in length, is the extracellular moiety of the M2 protein. This epitope is abundantly expressed on the surface of IAV-infected cells and M2e domains are highly conserved among IAV strains, making it an attractive target for a universal vaccine [12,13]. The protruding domain (P) particle platform is based on P particles which each contain 24 copies of Nv P domains with three exposed loops on the outermost surface in which the M2e epitope has been inserted [8]. The large complex-based platform is based on fusion of 2–3 dimeric/oligomeric antigens that are fused into one molecule through recombinant DNA technology with large, higher order complexes assembling spontaneously through dimerization and/or oligomerization of the homologous antigen [11,14]. Both P particle- (NvParticle/M2e) and large complex-based (NvComplex/M2e) vaccines containing the M2e epitope have been produced in *E*. *coli* with high yield and high stability. Mouse experiments revealed that both vaccines were highly immunogenic and were able to fully protect vaccinated mice against IAV lethal challenge using a mouse-adapted human IAV (H1N1, A/PR/8/34 [PR8] strain) [11,14].

The objective of this study was to determine the efficacy of a commercial inactivated pH1N1 vaccine administered intramuscularly and two experimental M2e subunit vaccines administered intranasally in protecting pigs from the effects of pH1N1 challenge.

## Materials and methods

### Ethical statement

The Iowa State University Institutional Animal Care and Use Committee approved this study (Approval number 3-16-8220-S). The efforts to alleviate suffering in this study included defined endpoints for each pig and independent veterinary supervision for the duration of the project.

### Pigs and experimental design

**Fig 1:**
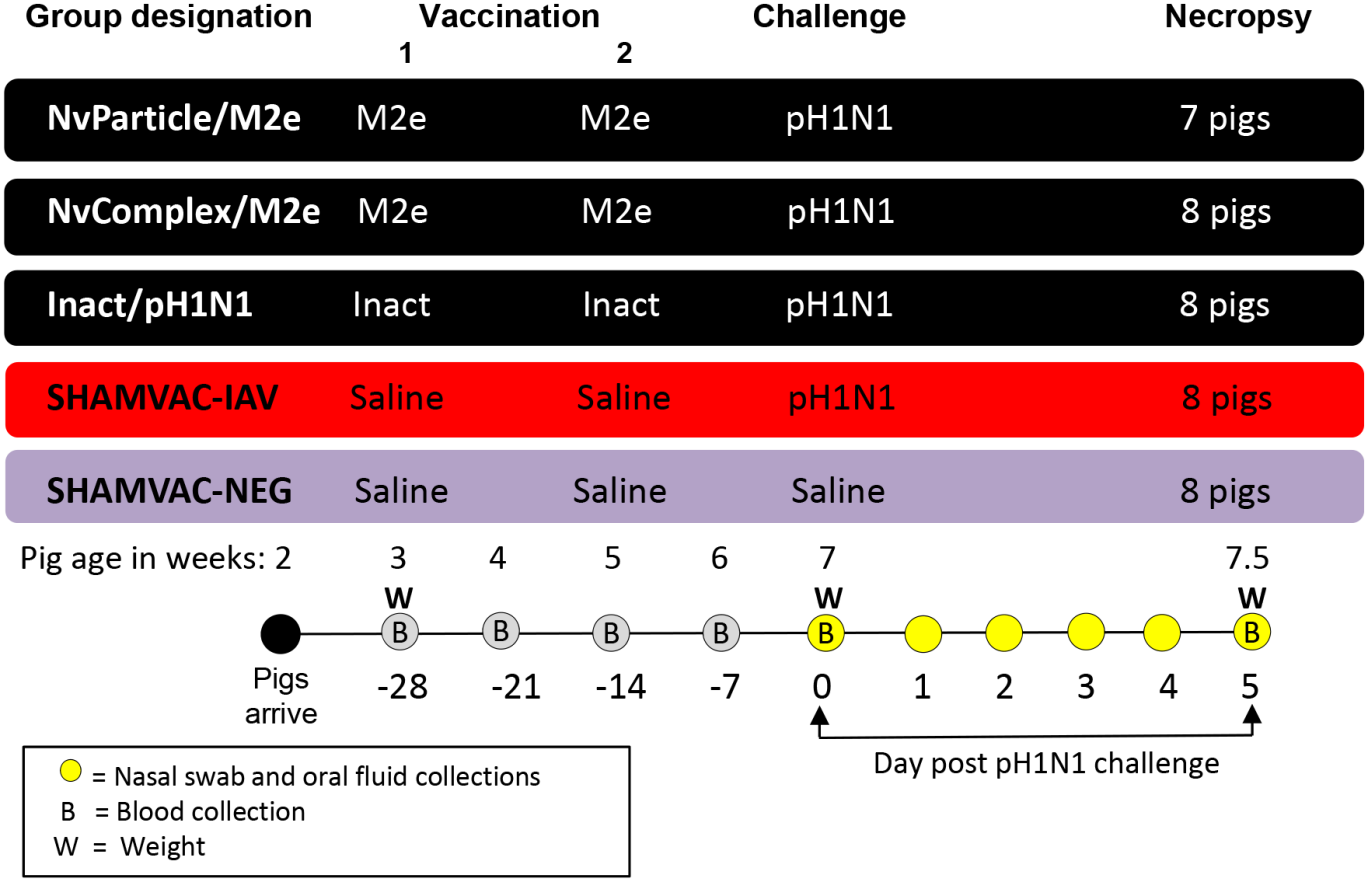
Experimental design. Vaccinations and sham-vaccinations were done when the pigs were 3 weeks old and repeated at 5 weeks of age. All pigs except the SHAMVAC-NEG group were challenged with IAV at 7 weeks of age. Necropsy was done at 5 days post challenge.

Thirty-nine 3-week-old pigs were obtained from an IAV-S-free herd and were randomly assigned to five rooms with 7–8 pigs in each group (Fig 1). Water and feed were provided *ad libidum*. Vaccination or sham-vaccination was done at 3 and 5 weeks of age followed by challenge or sham-challenge at 7 weeks of age. All pigs were euthanized 5 days post challenge (dpc). Blood was collected in serum separator tubes (Fisher Scientific, Pittsburgh, Pennsylvania, USA) weekly until challenge and at dpc 5. Blood tubes were centrifuged at 3000×g for 10 min at 4°C and serum was stored at -80°C until testing. Nasal swabs were collected individually from each pig on dpc 0, 1, 2, 3, 4 and 5 using polyester swabs and were stored in 5 ml plastic tubes containing 1 ml of sterile saline solution at -80°C until testing.

### Clinical assessment

All pigs were weighed at vaccination 1 (dpc -28), at challenge (dpc 0) and at necropsy (dpc 5) and the average daily weight gain was calculated. Rectal temperature, nasal discharge, cough and respiratory scores were assessed every day starting on dpc 0 (Fig 1). Nasal discharge was recorded as 0 = none, 1 = mild and 2 = severe and if present the location (left nostril, right nostril, both), color (clear, yellow, white) and consistency (watery, mucoid) were also noted. The cough score ranged from 0 = absent, 1 = single cough, and 2 = persisting cough, and respiratory scores ranged from 0 = normal to 6 = severe respiratory distress when at rest [15].

### Vaccines and vaccination

Vaccine details are summarized in Table 1. The vaccine used for the NvParticle/M2e group was a P particle-based vaccine [8,10] containing a pig IAV M2e epitope (SLLTEVETPIRNGWECKCNDSSD) [16]. The vaccine used for the NvComplex/M2e group was an agglomerate polymer-based (GST-P^+^) vaccine [11] containing a human IAV consensus M2e epitope (SLLTEVETPIRNEWGCRCNDSSD) [8,17]. Both vaccines were expressed in *E*. *coli*. The two M2e epitope sequences share 82% identity or 91% similarity. The vaccine used for the Inact/pH1N1 group was a commercially available inactivated IAV-S vaccine purchased from MWI Veterinary Supply (Boise, ID, USA).

**Table 1:** Details on the vaccines or sham vaccines used in the different treatment groups.

Group	Type	Antigen	Platform	Vaccination route
NvParticle/M2e	Subunit	M2e epitope	Nv P particle^3^	Intranasally
NvComplex/M2e	Subunit	M2e epitope	Nv Complex^4^	Intranasally
Inact/pH1N1	Commercial inactivated^1^	Whole pH1N1 virus^2^	NA^5^	Intramuscularly
SHAMVAC-IAV	Sham	Saline	NA	Intramuscularly
SHAMVAC-NEG	Sham	Saline	NA	Intramuscularly

^1^ FluSure^®^ Pandemic (Zoetis).

^2^ A/California/04/2009 (pH1N1).

^3^ Nv = norovirus, P = protruding domain. Each P particle contains 24 copies of NvV P domains with three exposed loops on the outermost surface in which the M2e epitope has been inserted.

^4^ Two to three dimeric/oligomeric antigens are fused into one molecule through recombinant DNA technology. The large, higher-order complexes of the fused antigens assemble spontaneously through dimerization and/or oligomerization of the homologous antigen.

^5^ Not applicable.

All pigs in the NvParticle/M2e group, NvComplex/M2e group and Inact/pH1N1group were vaccinated at dpc -28 and again at dpc -14. Pigs in the NvParticle/M2e and NvComplex/M2e groups were vaccinated intranasally with 2 ml of the P-particle vaccine or the complex vaccine, each containing 50 μg of protein vaccine by using a mucosal atomization device (MAD300, American Medical; Green Bay, WI, USA). While in previous mouse studies no adjuvants were used, in this study the vaccines were prepared by mixing 500 μl of the respective protein stock at a concentration of 1 mg/ml and 1.5 ml Polyinosinic-polycytidylic acid potassium salt adjuvant (PolyI:C; Sigma-Aldrich) at a concentration of 156 μg/ml shortly before administration to the pigs. Previously, PolyI-C adjuvant has been shown to enhance immune responses against IAV after intranasal administration [18]. Pigs in the Inact/pH1N1group were vaccinated with 2 ml of FluSure^®^ Pandemic (Zoetis; serial numbers 149390 and 120823) intramuscularly into the neck area after rehydration of the freeze-dried vaccine as recommended by the manufacturer.

### pH1N1 challenge

The pH1N1 isolate A/California/04/2009 was selected for challenge at dpc 0. The pigs were anesthetized with an intramuscular administered cocktail of ketamine (8 mg/kg of body weight), xylazine (4 mg/kg), and Telazol (6 mg/kg) as described [19] and challenged intratracheally (2 ml) and intranasally (1 ml) with the pdm09H1N1 strain at a dose of 2 × 10^5^ TCID_50_. Pigs in the SHAMVAC-NEG group were challenged by the same routes using similar amounts of saline.

### Serology

To assess the antibody response against IAV-S in serum samples, a commercial IAV-S blocking ELISA (IDEXX Swine Influenza Virus Ab Test; IDEXX Inc., Westbrook, MA, USA) was used according to the manufacturer’s instructions. This ELISA detects antibodies directed against the IAV nucleoprotein (NP). A sample-to-negative control (S/N) ratio less than 0.6 was considered positive. In addition to serum, this assay was also used on oral fluids and bronchoalveolar lavage (BAL) fluid with the following modifications: each plate was loaded with 200 μl undiluted OF or BAL fluid and incubated for 16 h at 21°C [20]. Serum, OF and BAL fluid reactions were measured as optical density (OD) at a wavelength of 650 nm using an ELISA plate reader. Sample-to-negative (S/N) ratios were calculated as described by the manufacturer, with S/N ratios of ≤0.60 considered antibody positive. For the OF test interpretation, in addition to the S/N ratio cut-off of ≤0.60 as suggested for serum samples by the manufacturer, results were also obtained and evaluated using a S/N ratio cut-off of ≤0.65 as suggested by a previous study [20]. In addition, serum samples collected at challenge (dpc 0) and necropsy (dpc 5) were also tested by a hemagglutinin inhibition (HI) assay. In brief, sera were heat inactivated at 56°C for 30 min to remove nonspecific hemagglutinin inhibitors and natural serum agglutinins, according to standard techniques used at the Iowa State University Veterinary Diagnostic Laboratory. The HI assay was performed using pH1N1antigen and turkey red blood cells.

### Demonstration of M2e specific antibodies

An *in-house* M2e ELISA to detect pig specific antibodies was performed as described elsewhere [10] and with modifications tested on lavage samples collected at necropsy for presence of anti-M2e IgA levels and on serum samples collected at challenge for anti-M2e IgG levels. Briefly, two synthetic M2e peptides, SLLTEVETPIRNGWECKCNDSSD for the NvParticle/M2e vaccine and SLLTEVETPIRNEWGCRCNDSSD for the NvComplex/M2e vaccine (3 μg/ml, Ohio peptide, USA), were used as detection antigens for M2e specific antibodies. Sera and lung washes were initially diluted to 1:25 (for lung washes) or 1:100 (for sera) and then 2-fold serial dilutions were made in 5% non-fat dry milk in PBS (pH 7.4) for the end-point titer determination. The M2e peptides were coated on 96 well microtiter plates (Nunc MaxiSorp^®^, USA) (200 ng/well) at 4°C for overnight. Before detection, the coated plates were blocked with 200 μl of 5% non-fat dry milk in PBS for 1 h at 37°C. After washing, diluted sera or lung washes (100 μl) were added to the plates for 1 h at 37°C. The bound antibody was detected by the HRP (horse reddish peroxidase)-conjugated goat anti-Pig IgG (for serum IgG) and HRP-conjugated goat anti-Pig IgA (for IgA in the lung wash) as secondary antibodies (Abcam, Cambridge, MA, USA). Antigen-specific antibody titers were defined as the endpoint dilution with a cut off signal intensity of 0.2.

Serum samples collected at the day of challenge were also characterized by western blot utilizing lysates from MDCK cells that had been infected with either PR8 virus or a PR8 7:1 reassortant with segment 7 from the pH1N1 strain A/California/ 07/2009 at an MOI of 5 as targets [21]. Cell lysates were harvested 24 hours post infection and run on SDS-PAGE gradient gels (4–20%) for separation of proteins before transferring to nitrocellulose membranes. Membranes were probed with a rabbit polyclonal anti-NP serum [21] to confirm infection and mouse monoclonal antibody 14C2 (Abcam) for detection of the PR8 M2 protein followed by rabbit and mouse secondary antibodies (1:20,000) as appropriate. Serum samples were tested using the Mini Protean II multiscreen (Biorad) with secondary swine antibodies (1:10,000). Membranes were developed by chemiluminescence using ECL Plus (Amersham).

### Detection and quantification of IAV-S specific nucleic acids and virus titration

Total nucleic acids were extracted from nasal swabs, oral fluids and bronchoalveolar lavage (BAL) samples using a MagMAX-96 viral isolation kit (Applied Biosystems, Life Technologies, Carlsbad, CA, USA) on a KingFisher^™^ Flex platform (ThermoFisher Scientific, Pittsburgh, PA, USA). Appropriate negative and positive controls were included in each extraction run. A quantitative reverse transcriptase (RT) real-time PCR based on the N1 gene specific for pH1 NA was performed on the RNA extracts. In addition, selected samples were also tested by a real-time PCR based on the M gene [22]. Virus titration was done on nasal swabs and BAL fluids as described [23] and titers were presented as TCID_50_ per ml.

### Necropsy and gross lung lesions

On dpc 5, all pigs were euthanized by intravenous pentobarbital overdose (FATAL-PLUS^®^, Vortech Pharmaceuticals LTD, Dearborn, MI, USA) and necropsied. The percentage of lung surface affected by visible lesions was assessed by a pathologist blinded to the pig treatment status using an established protocol [15]. BAL fluids were collected from each pig using minimal essential medium (MEM, Fisher Scientific). In addition, lung sections from the right cranial, right middle and accessory lung lobes and a section of distal trachea were collected. The sections were immersed in 10% neutral-buffered formalin and routinely processed for histopathology.

### Histopathology and immunohistochemistry

Tissue sections were fixed and microscopic lung lesions were assessed by a veterinary pathologist blinded to treatment status and scored for presence and degree of tracheitis, tracheal epithelial flattening or attenuation, bronchi and bronchiolar epithelial changes and peribronchiolar lymphocytic cuffing ranging from 0 = none to 4 = severe as described [23]. Presence and amount of IAV-S specific antigen in tissue sections were assessed by immunohistochemistry (IHC) as described [24,25]. The IAV antigen scores ranged from 0 = absent to 4 = abundant diffuse signals. Scoring was done blinded to the treatment status.

### Statistics

RNA genomic copies in nasal swabs and HI titers were log transformed prior to analysis. A one-way analysis of variance (ANOVA) was utilized to detect significant differences among treatment groups and pair-wise comparison were performed by least significant difference. The rejection level for the null hypothesis was *P* < 0.05. Pairwise testing was performed using Tukey-Kramer adjustment to identify different groups. Non-repeated measures were assessed using nonparametric Kruskal-Wallis ANOVA. When group variances were different, pair-wise comparisons were performed using the Wilcoxon rank sum test. Differences in incidence were evaluated by using Fisher’s exact test.

## Results

### Clinical disease

**Fig 2:**
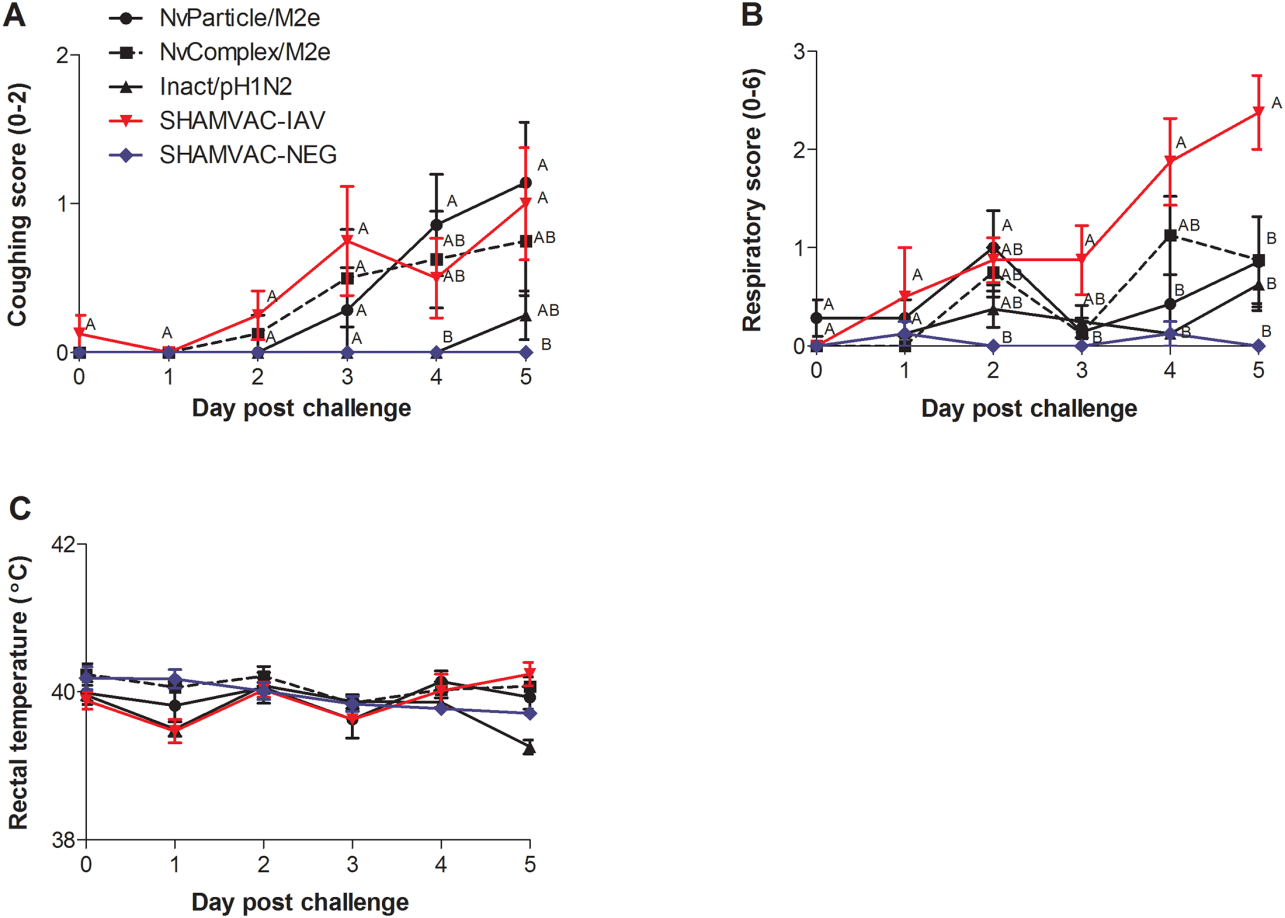
Mean group clinical assessment on the day of IAV challenge and on day post challenge (dpc) 1–5. **A**. Presence of cough ranging from 0 = absent to 2 = persisting cough. **B**. Respiratory score ranging from 0 = normal to 6 = severe respiratory distress when at rest. **C**. Rectal temperature. Different superscripts ^(A,B)^ on certain dpc indicate significantly (*P* < 0.05) different group means.

No clinical signs of respiratory disease were observed in any of the pigs prior to challenge or in the SHAMVAC-NEG pigs for the duration of the experiment. The average daily gain in grams ± SEM from the time of challenge until necropsy was 279.7±59.2 for the SHAMVAC-NEG pigs, 224.0±36.8 for the NvComplex/M2e pigs, 234.4±87.7 for the NvParticle/M2e pigs, 172.0±23.3 for the Inact/pH1N1 pigs and 207.9±54.6 for the SHAMVAC-IAV pigs which was not significantly different (*P* = 0.715). In IAV infected pigs, coughing was first observed in SHAMVAC-IAV and NvComplex/M2e pigs by dpc 2 (Fig 2A) in NvParticle/M2e pigs by dpc 3 and in Inact/pH1N1by dpc 5. Overall, 5/7 NvParticle/M2e pigs, 4/8 NvComplex/M2e pigs, 2/8 Inact/pH1N1pigs and 5/8 SHAMVAC-IAV pigs were observed coughing for 1–3 consecutive days and the average number of days with coughing was significantly higher for SHAMVAC-IAV pigs (1.5±0.4) compared to SHAMVAC-NEG pigs (0). Nasal discharge was observed in 4/7 NvParticle/M2e pigs, 5/8 NvComplex/M2e pigs, 2/8 Inact/pH1N1pigs and 8/8 SHAMVAC-IAV pigs for 1–3 days. The average number of days a pig was scored with a respiratory score of 1 or greater was 2.0±0.7 for NvParticle/M2e pigs, 1.8±0.5 for NvComplex/M2e pigs, 1.4±0.3 for Inact/pH1N1pigs, 3.3±0.4 for SHAMVAC-IAV pigs and 0.3±0.2 for SHAMVAC-NEG pigs. The group mean respiratory scores over time are summarized in Fig 2B and the mean group rectal temperatures are presented in Fig 2C. For the purpose of this study, fever was defined as a rectal temperature greater than 40.5°C. Two of 7 NvParticle/M2e pigs, 2/8 NvComplex/M2e pigs and 3/8 SHAMVAC-IAV pigs had fevers by dpc 4 and dpc 5 lasting 1–2 days without group mean differences among all groups.

### Serology

SHAMVAC-NEG pigs remained seronegative for IAV NP throughout the duration of the study (Table 2). At the dpc 0, 1/7 NvParticle/M2e and 1/8 Inact/pH1N1pigs had S/N ratios less than 0.6 and were considered positive. By dpc 5, the group mean Inact/pH1N1 ELISA value was significantly lower compared to all other groups which is indicative of seroconversion and 7/8 Inact/pH1N1 pigs were considered NP-seropositive based on the assay cutoff. In BAL fluid, 1/8 SHAMVAC-IAV pigs were ELISA positive (Table 2). Using a pH1N1 specific HI assay, SHAMVAC-NEG pigs remained seronegative for pH1N1 surface antigens throughout the duration of the trial (Table 2). Inact/pH1N1 pigs had significantly higher pH1N1 HI titers compared to all other groups by dpc 0 and 5. An *in house* assay was used to test for specific M2e IgA (BAL fluid) and IgG (serum samples) antibodies and a western blot assay was used to test for antibodies against the M2e PR8/Cal07 epitopes in serum samples. All samples were negative by the M2e IgA assay but IgG M2e specific antibody responses were detected in the NvParticle/M2e and the NvComplex/M2e pigs. Specifically, titers as high as 1:12800 were observed in1/7 NvParticle/M2e pigs and in 2/8 NvComplex/M2e pigs at the time of challenge. Moreover, the log transformed group mean in the NvComplex/M2e group was significantly higher compared to that of the SHAMVAC-NEG pigs (3.8±0.2 versus 3.5±0.1).

**Table 2:** Antibody responses as determined by a commercial blocking ELISA or a pdm09H1N1 specific HI assay on serum samples or bronchoalveolar lavage (BAL) fluid in the different treatment groups at -28, 0, and 5 days after pdm09H1N1 challenge.

Group	-28 (Serum)	0 (Serum)		5 (Serum)		5 (BAL fluid)
	*NP ELISA*^1^	*NP ELISA*^1^	*pH1N1 HI*^2^	*NP ELISA*^1^	*pH1N1 HI*^2^	*NP ELISA*^1^
NvParticle/M2e	0/7 (0.96±0.02)^A,^^3^	1/7 (0.94±0.06)^AB^	0/7 (3.1±1.9)^A^	0/7 (0.80±0.02)^A^	0/7 (3.8±1.5)^A^	0/7 (1.20±0.13)^A^
NvComplex/M2e	0/8 (1.00±0.01)^A^	0/8 (0.93±0.02)^AB^	0/8 (1.8±2.0)^A^	0/8 (0.83±0.03)^A^	0/8 (3.4±1.9)^A^	0/8 (1.29±0.12)^A^
Inact/pH1N1	0/8 (0.95±0.02)^A^	1/8 (0.74±0.08)^A^	8/8 (8.3±1.1)^B^	7/8 (0.42±0.05)^B^	8/8 (8.5±1.0)^B^	0/8 (0.88±0.05)^A^
SHAMVAC-IAV	0/8 (0.96±0.02)^A^	0/8 (0.94±0.04)^AB^	0/8 (1.5±1.8)^A^	0/8 (0.84±0.03)^A^	0/8 (4.2±1.5)^A^	1/8 (1.10±0.17)^A^
SHAMVAC-NEG	0/8 (0.94±0.01)^A^	0/8 (0.95±0.02)^B^	0/8 (2.2±2.0)^A^	0/8 (0.94±0.02)^A^	0/8 (2.1±1.8)^A^	0/8 (0.94±0.05)^A^

^1^ Positive pigs/total number of pigs per group (mean group sample-to-negative (S/N) nucleoprotein blocking ELISA ratios ± SEM). An S/N ratio less than 0.6 was considered positive.

^2^ Pigs with an HI titer > 40/total number of pigs per group [26] (mean log_2_ transformed pH1N1 HI titers ± SEM).

^3^ Different superscripts indicate significantly (*P* < 0.05) different group mean S/N values or HI titers at a dpc.

### Presence of IAV RNA

**Fig 3:**
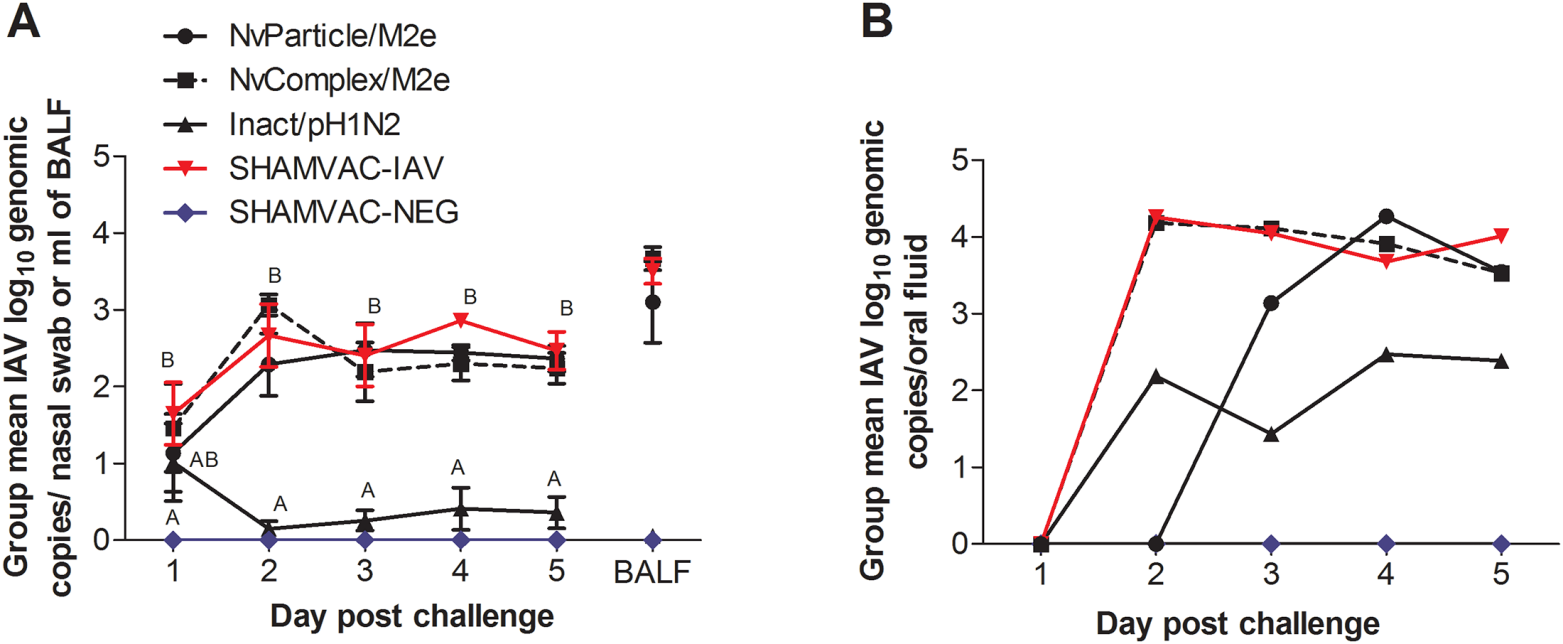
Mean group log_10_ IAV RNA genomic copies ±SEM. **A**. Nasal swabs were collected at day post challenge (dpc) 1–5 and bronchoalveolar lavage (BAL) fluid was collected on dpc 5. Different superscripts ^(A,B)^ on certain dpc indicate significantly (*P* < 0.05) different group means. **B**. Pen/group based oral fluid samples collected at dpc 1–5.

The amounts of IAV RNA in individual pigs are summarized for each group in S1 Fig. The group mean log_10_ IAV RNA levels in nasal swabs and BAL fluid are summarized in Fig 3A. IAV RNA was not detected in any of the SHAMVAC-NEG group samples and by dpc 2 mean group IAV RNA levels in nasal swabs and also in BAL fluids were significantly lower in Inact/pH1N1pigs compared to all other IAV infected groups. The group mean log_10_ IAV RNA levels in oral fluids are summarized in Fig 3B. There was a similar trend as seen with nasal swabs with Inact/pH1N1pigs having lower levels of IAV RNA in oral fluids compared to all other IAV infected pigs.

### Virus levels in nasal secretions and in lungs

In nasal swabs collected daily after challenge, virus was isolated from NvParticle/M2e pigs (5/7 on dpc 1, 0/7 on dpc 2, 3/7 on dpc 3, 2/7 on dpc 4 and on dpc 5), NvComplex/M2e pigs (5/8 on dpc 1, 5/8 on dpc 2, 2/8 on dpc 3, 2/8 on dpc 4 and 1/8 on dpc 4) and the SHAMVAC-IAV pigs (7/8 on dpc 1, 7/8 on dpc 2, 2/8 on dpc 3, 5/8 on dpc 4 and 4/8 on dpc 5) at certain days. At necropsy, 85.7% (6/7) of the NvParticle/M2e pigs, 50% (4/8) of the NvComplex/M2e pigs and 62.5% (5/8) of the SHAMVAC-IAV pigs had viable IAV virus in BAL fluids. All Inact/pH1N1pigs were negative for infectious IAV in nasal swabs and BAL fluids.

### Lesions and IAV antigen in tissue sections

**Fig 4:**
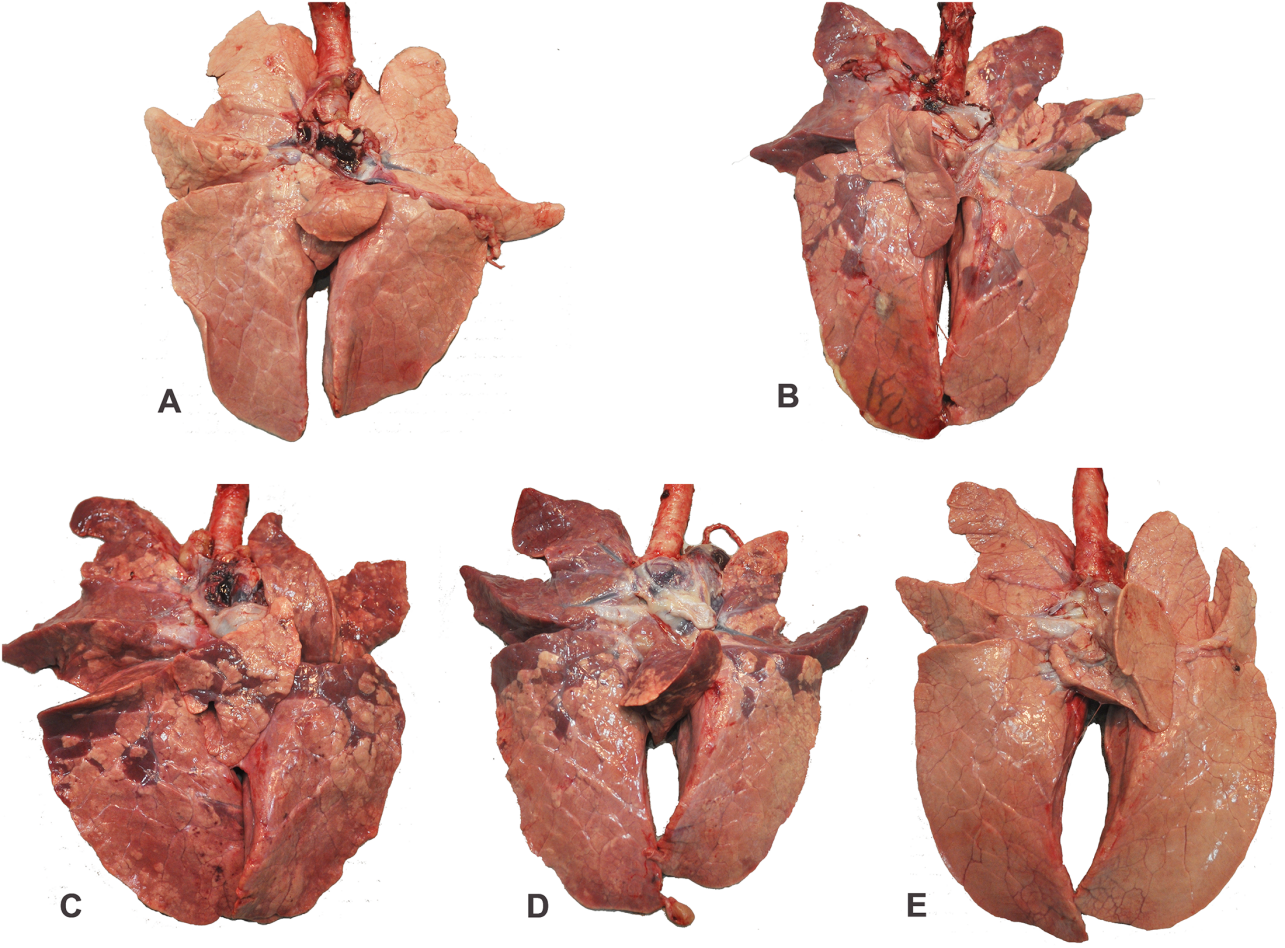
Macroscopic lung lesions at 5 days post IAV challenge. **A**. SHAMVAC-NEG pig 229: There are no visible lung lesions present (Score: 0). **B**. SHAMVAC-IAV pig 244: Dorsoventral there is diffuse severe dark purple consolidation present (Score: 30.1). **C**. NvParticle/M2e pig 239: Dorsoventral there is diffuse severe dark purple consolidation present (Score: 33.5). **D**. NvComplex/M2e pig 232: Dorsoventral there is diffuse severe dark purple consolidation present (Score: 31.1). **E**. Inact/pH1N1pig 234: There are no visible lung lesions present (Score: 0).

Macroscopic lung lesions associated with IAV infection were characterized by mild-to-severe, multifocal-to-diffuse, cranioventral, dark-purple consolidated areas (Fig 4); however, lesions were essentially absent in Inact/pH1N1pigs. Microscopically there was necrosis and flattening of the epithelium lining airways associated with accumulation of inflammatory cells in the lung and distal trachea. These lesions were almost always associated with large amounts of IAV antigen as determined by IHC stains (Table 3).

**Table 3:** Mean group macroscopic lung lesions (percentage of the lung surface affected by lesions) and prevalence and group mean microscopic lung lesions and IAV antigen as determined by immunohistochemical stains 5 days after pdm09H1N1 challenge.

Group	Macroscopic lung lesions	Microscopic lung lesions^1^ and IAV antigen			Microscopic tracheal lesions^1^ and IAV antigen		
		*Peribronchiolar cuffing*	*Epithelial necrosis*	*Lung IHC*^2^	*Tracheal epithelial flattening*	*Tracheitis*	*Trachea IHC*^2^
NvParticle/M2e	15.4±3.7^A,^^3^	6/7 (1.6±0.3)^A^	7/7 (2.1±0.2)^A^	7/7 (2.0±0.3)^A^	3/7 (0.7±0.4)^A^	5/7 (1.0±0.3)^A^	3/7 (0.6±0.3)^A^
NvComplex/M2e	14.3±3.7^A^	8/8 (2.6±0.4)^B^	8/8 (2.9±0.3)^A^	8/8 (2.8±0.3)^A^	5/8 (1.3±0.4)^A^	7/8 (1.3±0.4)^A^	8/8 (1.6±0.3)^A^
Inact/pH1N1	0.1±0.1^B^	1/8 (0.1±0.1)^C^	0/8 (0)^B^	0/8 (0)^B^	0/8 (0)^B^	0/8 (0)^B^	0/8 (0)^B^
SHAMVAC-IAV	11.8±3.2^A^	8/8 (1.8±0.3)^AB^	8/8 (2.3±0.3)^A^	8/8 (2.8±0.3)^A^	3/8 (1.0±0.3)^A^	5/8 (1.0±0.3)^A^	5/8 (0.6±0.2)^A^
SHAMVAC-NEG	0.1±0.0^B^	0/8 (0.1±0.1)^C^	0/8 (0)^B^	0/8 (0)^B^	0/8 (0)^B^	0/8 (0)^B^	0/8 (0)^B^

^1^ Score range from 0 = normal to 4 = severe.

^2^ Score range from 0 = not present to 4 = abundant.

^3^ Different superscripts indicate significantly (*P* < 0.05) different group mean values.

## Discussion

In this study, two novel experimental and broadly-reactive subunit IAV vaccines previously tested in mice [8–11] were compared to a commercially available conventional inactivated type-specific IAV vaccine in the pig model. In the past, experimental work carried out in mice has not translated well to humans limiting the usefulness of this model. The use of pigs models that are more similar to humans may be more appropriate [27].

Pigs vaccinated with the commercial inactivated type-specific vaccine were protected against pH1N1 challenge based on reduced shedding of IAV RNA in nasal secretions and oral fluids and reduced macroscopic and microscopic lesions; however, pigs vaccinated with the experimental broadly-reactive subunit vaccines were not, and developed clinical disease and severe macroscopic and microscopic lung lesions similar to the non-vaccinated SHAMVAC-IAV pigs.

Having access to a broadly reactive “universal” IAV vaccine would be a great asset to pig producers. Universal influenza vaccines have been reported previously, including self-assembling protein nanoparticles displaying M2e and Helix C with incorporation of TLR5 agonist flagellin, which has been shown to protect mice from lethal IAV challenge and also induced high levels of antibodies in chickens [28]. A novel M2e-tetra-branched multiple antigenic peptide based vaccine was evaluated in the mouse model and shown to be effective in protecting mice against heterologous strains [29]. Baculovirus constructs expressing HA fused to swine IgG2a Fc, displayed in a FeLV gag VLP, or displayed in the baculoviral envelope were generated and used to vaccinate pigs which elicited robust HI titers in serum and reduced lung lesions and prevalence of virus after challenge [30]. Previously, the same subunit vaccines tested in pigs in this study were used in the mouse model by vaccinating mice three times intranasally at 2-week intervals and using the mouse adapted H1N1 PR8 strain. The results demonstrated that the polyvalent GST-Nv P^−^-M2e vaccine fully protected mice against lethal challenge [14]. Similar results were also obtained with a GST-Nv P^+^-M2e polymer vaccine [11]. Despite being efficacious in mice using certain models, the vaccines failed to protect pigs in this study. This reinforces that findings in the murine model may not be predictive of responses in other animal species [31,32]. In addition, comparative mapping of anti-M2e antibodies obtained from different species revealed that one epitope was exclusively recognized by antibodies generated in rabbits while another epitope was only recognized by mice and chickens indicating differences in species recognition [33].

The previously observed protection of the two subunit vaccines in mouse trials and the lack of protection in this pig study potentially indicate differences in immune responses between species. The structural diversity and the abundance and distribution of nasal cavity-associated lymphoid tissues in both species may explain some of these differences [27,34,35]. Differences in immune responses were observed when comparing inbred (BALB/c, C57BL/6, C3H and BALB/cxC57BL/6) and outbred (CD1/ICR and Swiss Webster) mouse strains, since the second group exhibited poor anti-M2e Ab levels [36]. Another factor that may have influenced the immune response is the immunization route used herein. Most commercial pig vaccines available today are applied via the intramuscular route but it is suspected that, especially for respiratory infection, the intranasal vaccination would be beneficial. Therefore, the experimental M2e vaccines used in this study were given intranasally. Previous studies comparing intranasal versus subcutaneous administration routes in chickens and mice showed that subcutaneous immunization induced significantly higher Ab levels in the serum compared to intranasal [36,37] but we speculate that local mucosal protection against IAV is perhaps most important compared to systemic protection. Future studies need to directly compare both administration routes using the same products.

In addition, several factors regarding the design and application of the two subunit vaccines may also have contributed to the failure of protection observed in pigs in this study: (1) Unlike the inactivated whole pH1N1 virus-based Inact/pH1N1vaccine that should contain all original IAV epitopes, the 23 amino acid-M2e epitope of the two subunit vaccines represent only a small fraction of the IAV proteome. Hence, Inact/pH1N1 can elicit complex immune responses to inhibit various steps of IAV infection, while the subunit vaccines in this study did not elicit any detectable M2e-specific immune response. Previously it was found that M2e-based vaccines confer relatively weak protection mediated via non-neutralizing immune mechanisms compared to an inactivated IAV vaccine in mice [38]. Therefore, it may be understandable that the Inact/pH1N1vaccine exhibited much higher protection than the two subunit vaccines; however, studies comparing the Inact/pH1N1 vaccine to the experimental M2e vaccines in the mouse model are lacking. (2) Due to its small size, the M2e epitope constitutes only a small portion of the subunit vaccines, accounting for 7% and 4% of the NvParticle/M2e and the NvComplex/M2e total protein, respectively. As a result, higher vaccine dose and dosage than the currently studied ones may be required to induce a higher M2e-specific immune response. In previous trials, 15–30 μg of the subunit vaccines were used to immunize mice three times to show protection [10,11,14] and 50 μg of the same subunit vaccines for two immunizations may not have been sufficient to elicit protective immunity in the current pig study. Thus, future studies with higher doses of the subunit vaccine are necessary. (3) BLAST searches indicated that both M2e vaccine sequences used in this study match numerous M2e sequences in pig IAV strains recovered from the U.S. and other countries. For the particular pH1N1 challenge virus used in this study, both vaccines have a change on position 10 (Thr→Ile), and on positions 12 and 19 (Ser→Asn). In addition, the NvParticle vaccines has a change on position 13 (Glu→Gly) and on position 17 (Ala→Lys), while the NvComplex vaccine has a change on position 15 (Glu→Gly). While the M2e epitope has been considered to be very conserved posing an ideal universal IAV vaccine target [12,13], recognition of epitopes could have been affected by changes in amino-acid side chain polarity, as occurred with Thr→Ile on position 10 (both vaccines), and Glu→Gly on positions 13 (NvParticle vaccine) and 15 (NvComplex vaccine), due to conformation of the resulting protein. However, this needs to be further explored.

When tested with a commercial IAV NP-protein based blocking ELISA, most pigs in this study remained negative for IAV specific antibodies. Pigs vaccinated with experimental M2-based subunit vaccines were not expected to react with this assay since antibody directed against the NP protein do not cross react with those against the M2 protein. However, even in the Inact/pH1N1group only 1/8 pigs had a detectable response by dpc 0. Previously it has been determined that, while vaccination affected the S/N antibody response, data did not support the use of the NP ELISA for monitoring IAV vaccination compliance or for differentiating between IAV-infected animals and IAV-infected and vaccinated animals [26]. Because of this, an HI assay specific for subtype A/H1N1/pdm09 was also used. At challenge, all Inact/pH1N1pigs had titers of 80 or greater whereas in all other groups titers were between 0 and 20. This further confirms that all Inact/pH1N1pigs mounted a detectable immune response against pH1N1 by the time of challenge. Mucosal immunity specific for M2e was measured by IgA ELISA on lung lavage fluid collected at necropsy. However, none of the pigs had a detectable response. Nevertheless, results obtained with an IgG M2e ELISA on serum collected at challenge indicated a weak but detectable M2e-specific IgG antibody response in the NvParticle/M2e and the NvComplex/M2e pigs with significantly higher group means in the NvComplex/M2e pigs compared to the SHAMVAC-NEG pigs.

### Conclusions

Under the study conditions, pigs vaccinated with the commercial inactivated vaccine were protected against pH1N1 challenge based on reduced shedding of IAV RNA in nasal secretions and oral fluids and reduced macroscopic and microscopic lesions. Pigs vaccinated with the experimental subunit vaccines were not protected. Vaccine genetic makeup, dose, protein concentration and administration route need to be further investigated and better adjusted from usage in mice to usage in pigs.

## Supporting information

S1 Fig Individual pig log_10_ IAV RNA genomic copies in each group. Nasal swabs were collected at day post challenge (dpc) 1–5 and bronchoalveolar lavage (BAL) fluid was collected on dpc 5.
(TIF)

S2 Fig Minimal data set. (XLSX)
