# Supplementary figures and images for: Comparison of the efficacy of a commercial inactivated influenza A/H1N1/pdm09 virus (pH1N1) vaccine and two experimental M2e-based vaccines against pH1N1 challenge in the growing pig model
Source: PLoS One. 2018 Jan 30;13(1):e0191739. doi: 10.1371/journal.pone.0191739 (PMC5790244; doi:10.1371/journal.pone.0191739)

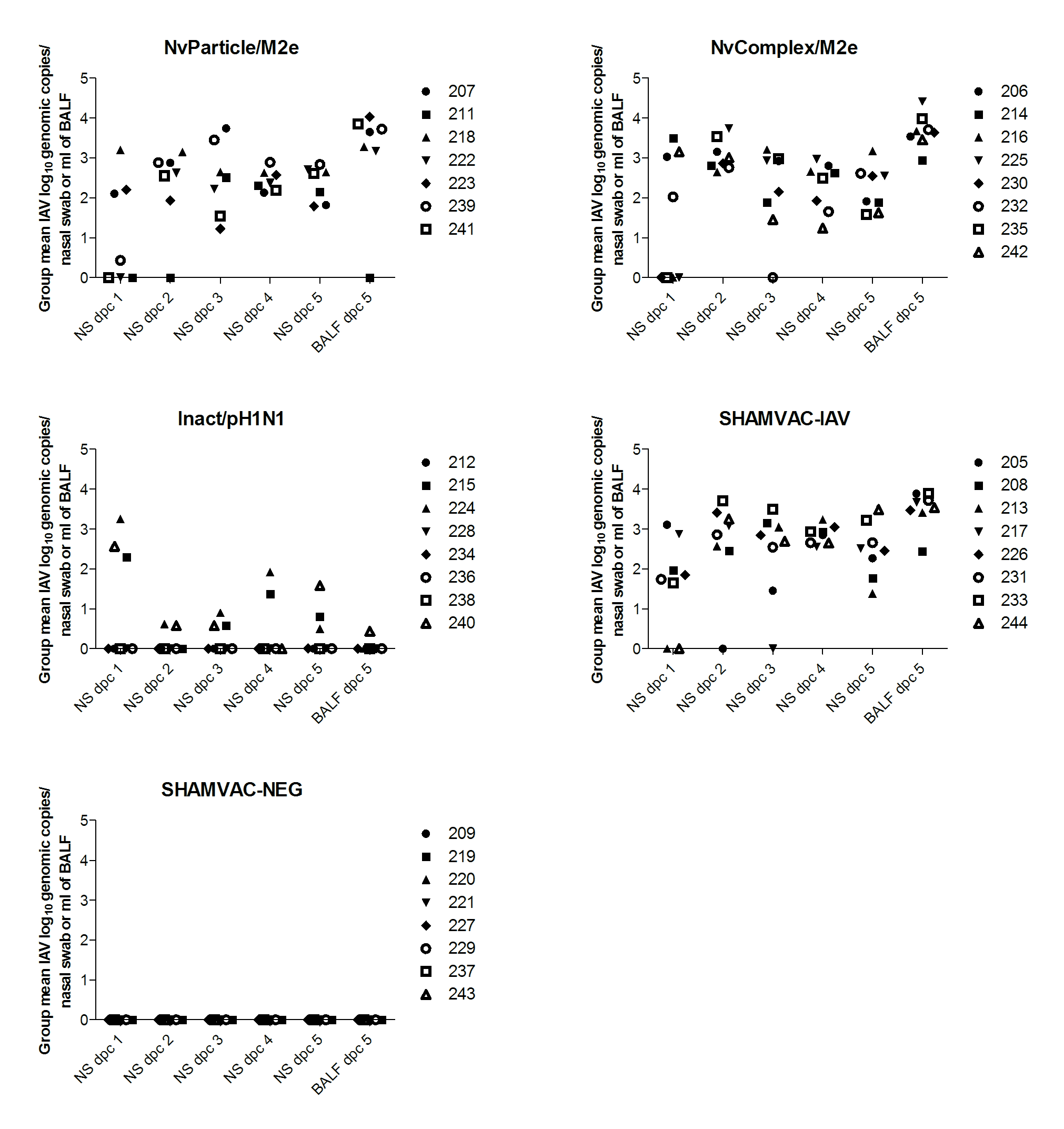

Supplement: S1 Fig — Nasal swabs were collected at day post challenge (dpc) 1–5 and bronchoalveolar lavage (BAL) fluid was collected on dpc 5. (TIF) [file pone.0191739.s001.tif]
